# Reassessing referral of touch following peripheral deafferentation: The role of contextual bias

**DOI:** 10.1016/j.cortex.2023.04.019

**Published:** 2023-10

**Authors:** Elena Amoruso, Devin B. Terhune, Maria Kromm, Stephen Kirker, Dollyane Muret, Tamar R. Makin

**Affiliations:** a Institute of Cognitive Neuroscience, University College London, London WC1N 3AZ, UK; b Department of Psychology, Goldsmiths, University of London, London SE14 6NW, UK; c Cambridge University Hospitals NHS Foundation Trust, Cambridge CB2 0QQ, UK; d Wellcome Trust Centre for Neuroimaging, University College London, London WC1N 3AR, UK

**Keywords:** Referred sensations, Amputees, Plasticity, Somatosensory cortex

## Abstract

Some amputees have been famously reported to perceive facial touch as arising from their phantom hand. These referred sensations have since been replicated across multiple neurological disorders and were classically interpreted as a perceptual correlate of cortical plasticity. Common to all these and related studies is that participants might have been influenced in their self-reports by the experimental design or related contextual biases. Here, we investigated whether referred sensations reports might be confounded by demand characteristics (e.g., compliance, expectation, suggestion). Unilateral upper-limb amputees (N = 18), congenital one-handers (N = 19), and two-handers (N = 22) were repeatedly stimulated with computer-controlled vibrations on 10 body-parts and asked to report the occurrence of any concurrent sensations on their hand(s). To further manipulate expectations, we gave participants the suggestion that some of these vibrations had a higher probability to evoke referred sensations. We also assessed similarity between (phantom) hand and face representation in primary somatosensory cortex (S1), using functional Magnetic Resonance Imaging (fMRI) multivariate representational similarity analysis. We replicated robust reports of referred sensations in amputees towards their phantom hand; however, the frequency and distribution of reported referred sensations were similar across groups. Moreover, referred sensations were evoked by stimulation of multiple body-parts and similarly reported on both the intact and phantom hand in amputees. Face-to-phantom-hand representational similarity was not different in amputees' missing hand region, compared with controls. These findings weaken the interpretation of referred sensations as a perceptual correlate of S1 plasticity and reveal the need to account for contextual biases when evaluating anomalous perceptual phenomena.

## 1 Introduction

Most amputees experience phantom sensations from their missing limb, typically described as itching, tingling, or numbness (Henderson & Smyth, 1948). These sensations typically manifest spontaneously but can sometimes also be triggered through stimulation of another body-part. Most commonly, phantom sensations can be evoked by touch applied to the residual limb (stump). This is believed to reflect peripheral reinnervation, where the severed sensory nerves, initially targeting e.g., the hand, reinnervate the surrounding tissue (Dhillon, Lawrence, Hutchinson, & Horch, 2004). A more curious example of evoked phantom sensations in upper-limb amputees comes from anecdotal reports that touching the face (e.g., while shaving) can elicit tingling sensations on the phantom hand (henceforth, “referred sensations”). In a famous series of case studies (Borsook et al., 1998; Halligan, Marshall, & Wade, 1994; Ramachandran, 1992, 1993), a small group of patients reported experiencing referred sensations from the ipsilateral face to the phantom hand. In some cases, the referred sensations were modality specific, with, for example, hot water applied to the face eliciting a warm sensation on the phantom hand. Strikingly, in most of these patients, neighbouring sites on the face elicited sensations on neighbouring fingers, suggesting a shared topographical organisation of the face and phantom hand.

Phantom referred sensations evoked by facial stimulation have been commonly interpreted as the perceptual correlate of primary somatosensory cortex (S1) remapping (Ramachandran, 1992). This was originally based on a classic electrophysiological study in non-human primates following long-term arm deafferentation, where the deafferented hand territory became responsive to touch applied on the monkey's chin (Pons et al., 1991). It is important to emphasise that deafferentation may lead to different consequences than amputation on peripheral reinnervation, and consequential physiological opportunities to stabilise the sensory system (Kuiken et al., 2007). Nevertheless, it was suggested that given a similar remapping process following amputation, face-induced activity in the missing hand area will be perceived as arising from the missing hand (hereafter, ‘the perceptual remapping hypothesis’) (Collins et al., 2017; Ramachandran, 1992, 1993; Ramachandran & Hirstein, 1998). This theory has been further extended to consider the neural origins of phantom limb pain (Flor et al., 1995), as well as analogous referred sensations described in other neurological disorders (Katz & Melzack, 1987; McCabe, Haigh, Halligan, & Blake, 2003; Moore et al., 2000; Soler et al., 2010).

However, subsequent studies using more systematic stimulation paradigms found that phantom referred sensations could be evoked by touches on various body-parts. This includes body-parts that have not been considered to invade the missing hand cortical area, such as the feet, trunk, and neck, in some cases even contralateral to the missing hand (Andoh et al., 2017; Grüsser et al., 2001, Grüsser et al., 2004; Halligan et al., 1994; Knecht et al., 1996, Knecht et al., 1998). These reports, as well as recent functional Magnetic Resonance Imaging (fMRI) studies that dispute the existence of a large-scale S1 facial remapping post-amputation in humans (Makin, Scholz, Henderson Slater, Johansen-Berg, & Tracey, 2015; Raffin, Richard, Giraux, & Reilly, 2016; Root et al., 2022; Valyear et al., 2020), challenge the perceptual remapping hypothesis.

An alternative mechanistic framework for understanding referred sensations is that previous studies, lacking adequate controls, might have been confounded by the demand characteristic which are typical of experimental settings where the desired outcome (or response) is known or can be implicitly inferred by the context (Orne, 1962). Beyond compliance effects, demand characteristics conveyed by a procedure or intervention can also produce genuine experiences. For example, verbal suggestions can elicit robust changes in perceptual states (Oakley, Walsh, Mehta, Halligan, & Deeley, 2021; Terhune, Cleeremans, Raz, & Lynn, 2017). It is increasingly recognised that demand characteristics in various forms may function as confounds in a variety of experimental paradigms (Foroughi, Monfort, Paczynski, McKnight, & Greenwood, 2016; Holman, Head, Lanfear, & Jennions, 2015; Lush, 2020; Szigeti et al., 2021; Van Dam et al., 2018). Therefore, rather than cortical plasticity, reports of referred sensations in previous experiments might be driven by both explicit suggestions from experimenters (e.g., “this procedure will produce this experience”) and implicit cues that promote expectations for specific experiences.

**Fig. 1:**
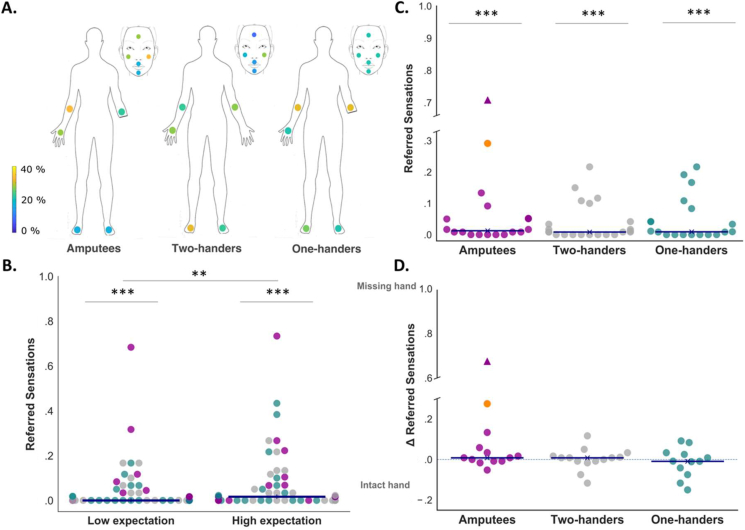
Global pattern of referred sensations across groups. A) Circles on the body silhouettes indicate the stimulated locations. The colour code indicates the percentage of participants in each group reporting at least one referred sensation at a given location. B) Proportion of referred sensations reported across groups in the Low and High expectation conditions. C) Proportion of referred sensations reported in each group across the stimulated locations. All groups reported referred sensations significantly above zero. D) Lateralised referred sensations across all stimulated locations, calculated by subtracting the proportion of referred sensations reported on the intact/dominant hand from the proportion of responses on the phantom/missing/non-dominant hand, in Amputees, Two-handers and One-handers, respectively. Positive values reflect referred sensations reported more towards the phantom/missing/non-dominant hand. Participants reporting zero referred sensations across the experiment were excluded (33.9% of the total sample). B–D) Each dot represents one participant; horizontal blue lines represent the group medians. Colours code for the different groups (see C and D). In C) and D) participant Amp05, who reported high rates of phantom referred sensations and who could be scanned (contrary to Amp07, triangle symbol), is highlighted in orange to ease qualitative comparison with fMRI results shown in Fig. 2. ∗∗∗*p* < .001, ∗∗*p* < .005, ∗*p* < .05.

**Fig. 2:**
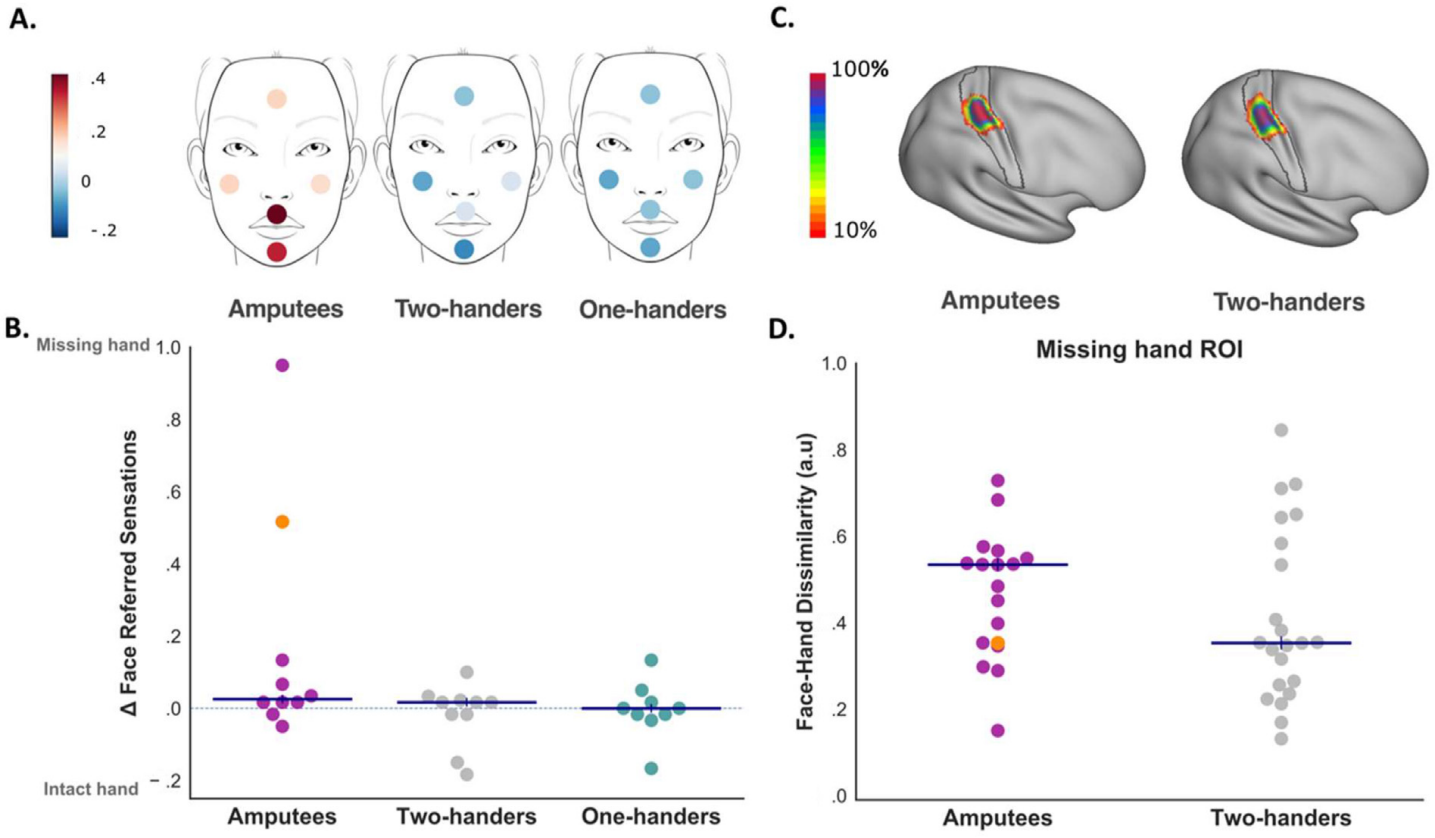
Face-evoked referred sensations and face-hand representational dissimilarity in the missing-hand region. A) Face locations stimulated in the referred sensations task. The colour code indicates the group median lateralisation scores of referred sensations from each location. B) Lateralised referred sensations across the five face locations (60 trials). Participants reporting zero referred sensations on any hand across the face trials are excluded (50.8% of total sample). C) Inter-participant consistency maps for the missing/non-dominant hand S1 regions of interest (ROIs) in Amputees and Two-handers. The colour code represents the number of participants with overlapping ROIs in standard Montreal Neurological Institute (MNI) space. The black contour shows the anatomical delineation of S1 used for ROI definition (see Supplemental information). D) Multivariate representational dissimilarity between activity patterns evoked in the missing/non-dominant hand ROI by facial (lips, nose, forehead) and contralateral thumb movements in Amputees and Two-handers (One-handers excluded as they cannot perform phantom movements). Whole samples are included (see Supplemental information for the same analysis in the sub-set of participants reporting referred sensations), each dot represents one participant, horizontal blue lines represent group medians. Participant Amp05 is highlighted in orange: despite reporting high rates of referred sensations, no decreased dissimilarity between face and phantom hand representations is observed.

Here we used vibrotactile stimulation [used to evoke reliable referred sensations in previous reports, e.g., (Grüsser et al., 2001; Knecht et al., 1996; Knecht et al., 1998)] of ten body-parts (previously reported to elicit referred sensations (Andoh et al., 2017; Grüsser et al., 2001; Grüsser et al., 2004; Halligan et al., 1994; Knecht et al., 1996; Knecht et al., 1998); Fig. 1, Fig. 2A) in a group of upper-limb amputees experiencing spontaneous phantom sensations. We also tested two control groups who do not report feelings of phantom sensations: two-handed individuals and individuals born with one hand. We manipulated participants' expectations for referred sensations with explicit verbal suggestions that specific vibro-tactile stimuli were more likely to evoke referred sensations on their hands (and even if that hand is missing). We predicted that if referred sensations reports are confounded by demand characteristics, then we should find similar reports across participant groups, and across the two hands in amputees. Alternatively, it is also possible that demand characteristics will be greater for a missing hand, leading to greater reports of referred sensations in both amputees and individuals born with one hand but only towards their phantom/missing hand. This is consistent with Bayesian models of perception, according to which the relative (precision) weighting of priors (e.g., expectations) in the emergence of perceptual states is greater when sensory evidence is imprecise. Finally, to evaluate the relation between referred sensations and cortical plasticity, we analysed functional neuroimaging (fMRI) data collected from two sensorimotor tasks in the same participants, using both univariate and multivariate approaches (Representational Similarity Analysis, RSA). We find that referred sensations can be generated and influenced by demand characteristics but no evidence to support the perceptual remapping hypothesis.

## 2 Methods

### 2.1 Participants

18 individuals with acquired unilateral upper-limb amputation (hereafter Amputees; mean age = 52 ± 12.2 (standard deviation, SD) y/o, 6 women, 12 men, 10 missing the right upper-limb; see Table 1 in Supplemental information for details about amputation, phantom limb pain and sensations), 19 individuals with congenital unilateral transverse arrest (hereafter One-handers; mean age = 44 ± 14.3 (SD) y/o, 11 women, 8 men, 7 missing right upper-limb), and 22 two-handed individuals (hereafter Two-handers; mean age = 45.5 ± 9.5 (SD) y/o, 10 women, 12 men, 6 left-handed) were tested. These samples size match the ones used in previous studies investigating remapping in these populations (e.g., (Makin et al., 2015; Root et al., 2022)). All One-handers reported that they did not experience any phantom sensations. One Amputee was not able to participate in the scanning session due to MRI safety concerns and another Amputee only completed the body task due to time constraints. The proportion of participants with intact/dominant right hand was matched between Amputees and both One-handers (*X*^*2*^_*(1)*_ = .942, *p* = .503) and Two-handers (*X*^*2*^_*(1)*_ = 2.670, *p* = .184). Amputees' gender was also matched to both One-handers (*X*^*2*^_*(1)*_ = 2.948, *p* = .106) and Two-handers (*X*^*2*^_*(1)*_ = 2.948, *p* = .106). Statistically significant differences for age were found between Amputees and One-handers (t_*(34)*_ = 2.280, *p* = .029) and Two-handers (t_*(37)*_ = 2.424, *p* = .020). Age covariates were therefore included when comparing neuroimaging observations between these groups. All participants provided written informed consent prior to participating in the study. No part of the study procedures or analysis plans was preregistered in an institutional registry prior to the research being conducted. We report how we determined our sample size, all data exclusions, all inclusion/exclusion criteria, whether inclusion/exclusion criteria were established prior to data analysis, all manipulations, and all measures in the study. The study was designed in accordance with the Declaration of Helsinki and was approved by the UK Health Research Authority (18/LO/0474).

### 2.2 Referred sensations task

#### 2.2.1 Procedure

Since demand characteristics are incredibly difficult to avoid in experimental settings involving subjective reports (Boot, Simons, Stothart, & Stutts, 2013; Klein et al., 2012; Szigeti et al., 2021; Turner, Deyo, Loeser, Von Korff, & Fordyce, 1994), and since the notion of referred sensations in amputees is very popular (Ramachandran & Blakeslee, 1998), we manipulated participants' explicit expectancies. This procedure is commonly used in suggestion studies (Alganami, Varese, Wagstaff, & Bentall, 2017; Crichton et al., 2014; Sørensen, Vangkilde, & Bundesen, 2015) and allowed us to minimise any group differences due to prior beliefs, experiences and expectations relating to referred sensations.

Participants sat in front of a computer screen (2560 × 1440, 60 Hz) and a pedal was positioned underneath each foot for response collection. Vibrating motors (diameter: 10 mm, thickness: 2.7 mm, operating voltage: 5.5 V DC) were secured with surgical tape on ten body-parts (Fig. 1, Fig. 2A). Participants were informed that some of the vibrations were stimulating a newly discovered type of afferent fibres that could evoke sensations not only on the stimulated body-part but also on other areas, most commonly on the hands (see Supplemental information for verbatim instructions). Participants were told that a red or grey circle display would indicate whether an incoming stimulus had high or low probability, respectively, of evoking these dual sensations (hereafter, “High” and “Low” expectation conditions). All stimuli were, in effect, simple 500 msec vibrating trains. To induce the feeling that vibrations may vary, and thus increase the belief in the suggestion, trains were delivered at an intensity well above detection threshold at three frequencies (15 Hz, 12 Hz, or 9 Hz), with equal distribution across the different locations and cues. Participants were instructed to stay still and to focus on potential sensations arising from their hands.

Except for the instruction phase, the experiment ran in a strictly automated manner, with the experimenter leaving the room and visual cues, stimuli and responses delivered and collected by the software (MATLAB r2017a). Each trial began with the red/grey visual cue displayed for 700 msec, followed by the 500 msec vibration on one body-part. Next, a question appeared on the screen: “Have you just felt one stimulus or more than one?” Participants were instructed to press with the left foot to respond “one” and with the right to respond “more than one”. When reporting multiple sensations, participants were further asked to respond, with the corresponding foot pedal, to the question: “Was it on the right or the left hand?”. Amputees and One-handers were briefed that this question also related to their phantom/missing hand (respectively). The experiment included 120 trials, equally distributed across the 10 stimulated locations, 60 trials per expectation cue (High/Low), and 40 trials per vibration frequency (9 Hz, 12 Hz, 15 Hz). Due to technical issues during data collection, two Amputees, one One-hander and one Two-hander performed a reduced number of trials (90). At the end of the session participants were debriefed about the deception.

#### 2.2.2 Analyses

Trial-level data were aggregated into participant-level data by calculating the proportion of reported referred sensations in each condition. To focus on referred sensations reported on the phantom hand, while accounting for responses on the intact hand, lateralisation scores were calculated by subtracting the proportion of intact/dominant hand to phantom/missing/non-dominant hand referred sensations reports, in Amputees, One-handers and Two-handers respectively. Only participants reporting at least one referred sensation in a given condition of interest were included in this calculation. This resulted in different sample sizes across analyses/groups (see Results).

### 2.3 Functional MRI tasks

#### 2.3.1 Procedure

The scanning session was completed on the same day as the behavioural task, apart from one Amputee who was scanned only 2 years later (due to Covid-19 restrictions; Amp10 in Table 1). In brief, the conditions included in the present analysis involved visually-instructed body-part movements.

For the body task (used as functional localiser in (Muret, Root, Kieliba, Clode, & Makin, 2022)), participants were visually instructed to move one of five body-parts (see Fig. S2): intact/dominant hand (for participants with a missing hand and Two-handers, respectively; opening and closing the hand), residual/non-dominant arm (flexing the most distal residual joint for participants with a missing hand and the elbow for Two-handers), right or left toes (wiggling the toes) or lips (puckering the lips). An additional condition involving the missing/non-dominant hand was also included but will not be further described as it was not included in the present analyses. Movements were repeated at a constant instructed pace for a period of 12 sec, interleaved with 12 sec of rest. Each condition was repeated 4 times in a pseudo-random order.

For the face task, the full details of the procedures and acquisition parameters can be found in (Root et al., 2022). In short, participants were instructed to perform one of five movements: raise the eyebrows (i.e., forehead), flare nostrils (i.e., nose), puckering lips (i.e., lips), and flex the left or right thumb (or phantom thumb, if available). When phantom sensations were not present, participants with a missing hand were asked to imagine performing the movement. Note that this dataset was used to determine the relationship between the phantom hand and the face representation, and for this reason we did not include the congenital one-handers in this analysis. An additional condition involved tapping the tongue to the roof of the mouth. However, since the inner mouth was not investigated in our behavioural task, we excluded this condition from our analysis in the present study. Instructions and pace were provided visually via a screen, resulting in 5 cycles of movement per 8 sec block. Each movement block was repeated 4 times per run, which also comprised 5 blocks of rest used as baseline.

Conditions were pseudo-randomly distributed, such that each condition was equally preceded by all other conditions. Prior to entering the scanner room, participants were thoroughly instructed, and all movements were practiced in front of the experimenter to ensure they were performed correctly. To confirm that appropriate movements were made at the instructed times, whenever possible – task performance was visually monitored online for both tasks. These datasets were recently used for other purposes [i.e., body task used as functional localiser in (Muret et al., 2022) and face task analysed in more detail in (Root et al., 2022)], but the body dataset was not used before to assess remapping.

#### 2.3.2 MRI data acquisition

MRI images were acquired using a 3T Prisma MRI scanner (Siemens, Erlangen, Germany) with a 32-channel head coil. Functional data were obtained using a multiband T2∗-weighted pulse sequence with a between-slice acceleration factor of 4 and no in-slice acceleration. The following acquisition parameters were used: Repetition Time (TR) = 1450 msec; Echo Time (TE) = 35 msec; flip angle = 70°; voxel size = 2 mm isotropic; imaging matrix = 106 × 106; Field of View (FOV) = 212 mm. 72 slices were oriented in the transversal plane covering the entire brain. Each dataset comprised one and three functional task-related block-design runs (for the body and face tasks respectively). Field-maps were acquired for field unwarping. A T1-weighted sequence (Magnetization Prepared RApid Gradient Echo (MPRAGE), TR = 2530 msec; TE = 3.34 msec; flip angle = 7°; voxel size = 1 mm isotropic) was used to obtain anatomical images.

#### 2.3.3 Functional MRI data pre-processing and analysis

Functional data was analysed in individual's native functional space and pre-processed in FSL-FEAT (v6.00). Pre-processing included the following steps: motion correction using MCFLIRT (Jenkinson, Bannister, Brady, & Smith, 2002); brain extraction using Brain Extraction Tool (BET) (Smith, 2002); high-pass temporal filtering with a cut-off of 280 sec and 119 sec for the body and face task respectively; and finally spatial smoothing using a Gaussian kernel with a full width at half maximum of 5 mm and 3 mm for the body and face task respectively. Field maps were used for distortion correction. For the face task, a midspace between the different functional runs was calculated for each participant, i.e., the average space in which the images are minimally reorientated. Each functional run was then aligned to the midspace and registered to each individual structural T1 scan using FMRIB's (Oxford Centre for Functional Magnetic Resonance Imaging of the Brain) Linear Image Registration Tool (FLIRT), optimised using Boundary-Based Registration (Greve & Fischl, 2009).

We focused on the S1 hand region, though marginal contribution from M1 may have affected activity profiles due to its spatial proximity. The S1 hand region of interest (ROI) was defined bilaterally for each individual on a template surface using probabilistic cytoarchitectonic maps, by selecting nodes showing maximal probability for the grey matter of Brodmann areas (BAs) 3a, 3b, 1 and 2 see Root et al. (2022), approximately 1 cm below and above the hand knob. This criterion defined a more conservative hand region than in previous research (Kieliba, Clode, Maimon-Mor, & Makin, 2021; Wesselink et al., 2022; Wiestler & Diedrichsen, 2013), in order to minimise overlap with the neighbouring face/arm areas. Structural T1-weighted images were used to reconstruct the pial and white-grey matter surfaces using Freesurfer. Surface co-registration across hemispheres was done using spherical alignment. The anatomical hand ROIs were projected into the individual brains via the reconstructed individual anatomical surfaces. For visualisation (Fig. 2C), S1 ROIs of each participant were projected to Montreal Neurological Institute (MNI) 152 space using the nonlinear registration carried out by FMRIB’s Nonlinear Image Registration Tool (FNIRT). Participant information regarding the side of missing/non-dominant hand were used to sagittal-flip data, such that the ROIs contralateral to the missing hand were always represented in the right hemisphere. ROIs of all participants were then concatenated into a single volume to produce a consistency map (i.e., how many participants have their ROIs overlapping in the MNI space). Resulting consistency maps were then projected to a group cortical surface^56^ using Connectome Workbench (v1.4.2). For univariate analysis (see Supplemental material), the z statistic timeseries from all voxels of each ROI obtained for each movement were extracted and averaged.

#### 2.3.4 Multivariate representational similarity analysis

The dissimilarity between activity patterns generated by the contralateral thumb and the four face parts within each S1 hand ROI was computed at the individual level for each pair of movements using cross-validated squared Mahalanobis distance (Walther et al., 2016). As One-handers do not have a representation of their missing hand, they were excluded from this analysis. Multidimensional noise normalisation was used to increase reliability of distance estimates (noisier voxels are down-weighted), based on the voxel's covariance matrix calculated from the General Linear Model (GLM) residuals. Due to cross-validation, the expected value of the distance is zero (or negative) if two patterns are not statistically different from each other, and significantly greater than zero if the two representational patterns are different (Diedrichsen, Provost, & Zareamoghaddam, 2016). Larger distances for movement pairs therefore suggest greater information content. The resulting representational pairwise distances between each of the facial conditions and the thumb (phantom/nondominant and intact/dominant, in Amputees and Two-handers respectively) were extracted. The analysis was conducted on an adapted version of the RSA Toolbox in MATLAB (Nili et al., 2014), customised for FSL (Wesselink & Maimon-Mor, 2018).

### 2.4 Statistical analyses

All statistical analyses were carried out using JASP (v0.14.1). To identify violations of the normality assumption, Shapiro–Wilk tests were run. No outliers were removed from the analyses to not exclude individuals showing potentially high referred sensations rates, large remapping or face-hand representational dissimilarity. For non-significant comparisons of interest, Bayesian *t*-tests (or non-parametric equivalents) were conducted, with a Cauchy prior width set to .707 (default). We report Bayes Factors (BF_10_), showing the relative support for the alternative hypothesis (Dienes, 2014). As measures of effect size, rank–biserial correlations (r_B_) or Cohen's ds are reported. Kendall's Tau correlations were used to investigate whether referred sensations reports or cortical remapping were related to phantom limb pain (PLP) in Amputees.

For the behavioural task, as normality was consistently violated across conditions, nonparametric tests (i.e., Wilcoxon signed-rank, Mann–Whitney, and Kruskal–Wallis tests) were used to test for within- and between-subject differences in the overall proportions and lateralisation of reported referred sensations.

fMRI data was analysed using mixed ANOVAs with the between-subject factor of Group (three groups) and a repeated-measure factor of Hemisphere (intact/dominant x deprived/non-dominant), and age as a covariate to account for age differences in cortical activation. If assumptions of normality were violated, non-parametric equivalents are also reported. Post-hoc comparisons between groups were conducted with a Bonferroni correction for multiple comparisons (*α* = .025; uncorrected *p*-values reported in the text). To identify which body-parts were driving the observed remapping in the missing-hand region, independent samples *t*-tests were used to assess group differences between one-handed groups and Two-handers, using Bonferroni correction of alpha levels (*α* = .01) to account for comparisons across the five body-parts.

## 3 Results

### 3.1 When sharing similar expectations, amputees do not report more referred sensations than one- and two-handers

First, we examined if Amputees are more prone to reporting referred sensations, relative to One- and Two-handers. We found that all groups reported experiences of referred sensations significantly above zero (Z ≥ 78, *p* < .005, r_B_ = 1.0, for all groups) with no significant differences in the proportion of trials where referred sensations were reported across three different stimulation frequencies (*X*^*2*^ ≤ 2.311, *p* ≥ .315 for all groups). This result demonstrates that, when placed in a similar state of expectancy towards sensations on the hands, many diverse samples irrespective of amputation, can report referred sensations. Across groups, trials in the High expectation condition evoked more reports than in the Low expectation condition (N = 59, Z = 206.5, *p* = .047, r_B_ = −.38) (Fig. 1B), indicating that referred sensations can be enhanced through explicit suggestions. We observed no group differences for this suggestion effect (*X*^*2*^ = .560, *p* = .756), with Amputees' difference (High – Low) scores not different from One- and Two-handers (both U > 173, *p* > .499, r_B_ < −.124, BF_10_ < .403). Interestingly, referred sensations were reported more frequently relative to zero across all groups even during the Low expectation condition (N = 59, Z = 351, *p* ≤ .001, r_B_ = 1.0; 42.4% of participants reported >0 referred sensations), plausibly reflecting an attentional effect wherein the instruction to attend to specific body-parts elicits reports of sensations that would otherwise pass unnoticed or perceptual false positives (Brown et al., 2012; Mirams, Poliakoff, Brown, & Lloyd, 2012). For this reason, further analyses were based on the proportion of referred sensations collapsed across expectancy conditions. Crucially, the overall proportion of reported referred sensations did not differ across groups (*X*^*2*^ = .338, *p* = .845), and Amputees (N = 18) did not report more referred sensations than One- (N = 19) or Two-handers (N = 22) (both U < 220, *p* > .550, r_B_ < .111, BF_10_ < .355) (Fig. 1C). In summary, participants of all groups responded positively, but similarly, to the suggestion cues, regardless of amputation, but to a greater extent in the high versus low expectation condition.

Given the presumed mechanistic relationship (i.e., brain plasticity) between referred sensations and phantom limb pain (PLP) (Flor et al., 1995, 2013) (though see also (Makin et al., 2013)), we also explored the correlation between the propensity to report referred sensations on the phantom hand (while accounting for intact hand reports) and chronic PLP (Table 1), and found no significant correlation and Bayesian evidence for a null correlation (N = 18, r_Tau_ = .074, *p* = .692, BF_10_ = .327).

### 3.2 Sensations are not differentially referred to amputees' phantom hand

Next, we assessed the specificity of Amputees' phantom hand as a target for referred sensations (Fig. 1D). Both One- (N = 12) and Two-handers (N = 14) reported similar proportions of referred sensations across the two hands (both Z ≤ 53.5, *p* ≥ .583, r_B_ ≤ −.192, BF_10_ ≤ .353). If referred sensations result from deprivation-triggered cortical remapping, then they should occur more frequently on Amputees' phantom hand. However, we found no significant differences in the proportion of reported sensations across the two hands in Amputees (N = 13; Z = 59, *p* = .125, r_B_ = .513, BF_10_ = 1.11), or in the lateralisation towards the missing/non-dominant hand across groups (*X*^*2*^ = 2.498, *p* = .287). Amputees did not show greater lateralisation towards the phantom hand than One- or Two-handers (both U ≥ 106, *p* ≥ .134, r_B_ ≤ .359, BF_10_ ≤ .940), indicating that they were not more inclined to report referred sensations on the phantom hand than One- and Two-handers on their missing/non-dominant hand, respectively. Finally, no differences were observed between One-handers' and Two-handers' lateralisation scores (U = 70, *p* = .486, r_B_ = −.167, BF_10_ = .417).

### 3.3 Face-evoked referred phantom sensations are not reflected in shared S1 representation

According to the “perceptual remapping hypothesis” (Collins et al., 2017; Halligan et al., 1994; Ramachandran, 1992, 1993; Ramachandran & Hirstein, 1998), phantom hand referred sensations in amputees should more likely occur following face stimulation, due to the proximity in S1 representations. In accordance with this hypothesis, we found that face-evoked referred sensations (Fig. 2A) were reported marginally more frequently on the phantom/missing/non-dominant rather than on the intact/dominant hand in Amputees (N = 10) (Z = 46.5, *p* = .058, r_B_ = .691, BF_10_ = 2.215), but not in One-handers (N = 9) and Two-handers (N = 10) (both Z ≤ 30, *p* ≥ .836, r_B_ ≤ .091, BF_10_ ≤ .330) (Fig. 2B). However, no significant group difference emerged (*X*^*2*^ = 3.417, *p* = .181), with Amputees' phantom lateralised face-evoked referred sensations not significantly different from One-Handers' (both U ≤ 69.5, *p* ≥ .109, r_B_ ≤ 444, BF_10_ ≤ .952), and no significant differences between One-handers and Two-handers (U = 41.5, *p* = .805, r_B_ = −.078, BF_10_ = .414) (Fig. 2B), which could be attributed to reduced statistical power, as indicated by the Bayes factors (see also Fig. S1 for an analysis of referred sensations evoked by other stimulation sites).

To more directly test the idea of shared representation between the face and phantom hand representations (as well as other body-parts), we took advantage of two fMRI datasets that have been collected in the current study sample. Using a motor task involving movement of different body-parts, we replicated the well-known patterns of S1 body-part remapping across the three groups, which are qualitatively, though clearly, distinguishable from the even inter-group profile observed for the referred sensations reports (Figs. S2 and S3). Nevertheless, this approach relies on net activity levels within the missing hand region, while disregarding the well-established finding that the representation of the phantom hand persists in amputees (Makin & Bensmaia, 2017; Bruno, Ronga, Fossataro, Capozzi, & Garbarini, 2019; van den Boom et al., 2021; Garbarini, Bisio, Biggio, Pia, & Bove, 2018; Gagné, Hétu, Reilly, & Mercier, 2011). Thus, it is still possible that deprivation-triggered plasticity elsewhere along the somatosensory pathway (e.g., brainstem (Kambi et al., 2014), thalamus (Jain, Qi, Collins, & Kaas, 2008)) generates a neural scaffolding for shared representation between the phantom hand and other body-parts, which may not be easily observed using an univariate mapping approach (Muret et al., 2022; Wesselink et al., 2022). If referred sensations are associated with deprivation-triggered plasticity along the sensorimotor pathway, this should therefore result in greater representational similarity between amputees' face and phantom hand, relative to two-handed controls (or to amputees' own intact hand representation in their intact hand region). To test this hypothesis, the multivariate representational dissimilarity between activity patterns evoked by face and contralateral thumb were compared in the hand region across hemispheres and groups (see Fig. 2D for the deprived hemisphere). To generate a representation of the phantom hand, participants were required to actively move their (phantom) thumbs, as well as facial sub-parts (Root et al., 2022). No significant differences were found between Amputees and Two-handers (F_(1,36)_ = .372, *p* = .546, *η*^2^ = .009), and no interaction with the hemisphere (F_(1,36)_ = 1.402, *p* = .244, *η*^2^ = .004). Follow-up comparisons revealed no significant difference between hemispheres in Amputees (t_(16)_ = .619, *p* = .545, d = .150, BF_10_ = .295), as well as no group difference in the dissimilarities observed in the missing/non-dominant hand region (t_(37)_ = −1.047, *p* = .302, d = −.338, BF_10_ = .483). In other words, we did not find any evidence for shared information content between the phantom hand and the face in the missing hand region (see (Root et al., 2022) for further characterisation of the lack of facial remapping in this cohort of participants).

## 4 Discussion

Here, we studied referred sensations reports in a group of upper-limb amputees experiencing spontaneous phantom limb sensations, in comparison to two control groups who had not undergone amputation (congenital one-handers and two-handers). Amputees did not exclusively refer these sensations to their phantom hand and did not report more induced referred sensations than the control groups. In all groups, referred sensations could be evoked from stimulation of multiple body-parts on both sides of the body, irrespective of inter-group differences in S1 remapping (Figs. 1, S2, S3; see also (Andoh et al., 2017; Flor et al., 2000)). Our results demonstrate that, when placed in a similar state of expectancy towards sensations on the hands, many diverse samples, irrespective of amputation, can report referred sensations. The high response rates, observed even under low expectancy settings, might also reflect the idea that referred sensation are naturally occurring, irrespective of amputation (e.g., due to crossing effects of touch or overweighting of incoming sensory evidence (Badde, Röder, & Heed, 2019)). While both explanations are likely to play a role in our results, they empirically challenge the hegemonic view that referred sensations are a specific consequence of amputation.

When sensations were evoked by face stimulation, we found marginal evidence for amputees differentially referring these sensations to the phantom hand, though this did not drive a significant interaction across groups. Importantly, as we show here using representational similarity analysis, this marginal effect in amputees is likely not consequential to increased sharing of S1 resources between the face and the phantom hand in the missing-hand region (Fig. 2D; see also (Makin et al., 2015; Raffin, Pellegrino, Di Lazzaro, Thielscher, & Siebner, 2015; Root et al., 2022; Valyear et al., 2020) for evidence of minimal face remapping). When comparing the similarity between the face and phantom hand representations in amputees we find no difference between the two hemispheres (as confirmed with a Bayes Factor showing substantial evidence in favour of the null hypothesis), as well as no significant differences between hand-to-face similarity between amputees and controls. This evidence provides further support for the accumulating body of evidence (using behavioural, neuroimaging, electrophysiological and transcranial magnetic stimulation studies (Brown et al., 2012; Flor et al., 2013; Makin et al., 2013; Mirams et al., 2012), see (Makin & Bensmaia, 2017) for a review) that (phantom) hand representation is relatively invariant following amputation.

In this context, it is important to note that the only methodology we could harness to reliably trigger phantom hand representation which could be compared across participants and groups involves active movement. While recent studies emphasise that representational structure in S1 is comparable across passive and active stimulation of the hand (see (Berlot, Prichard, O’Reilly, Ejaz, & Diedrichsen, 2019; Sanders et al., 2023); see (Root et al., 2022; Valyear et al., 2020) for related findings for the face), it is important to note that referred sensations are predominantly associated with touch, and not movement. As we did not attempt to evoke referred sensations in our fMRI paradigm, our neuroimaging findings are not ideally suited to uncover the neural basis of referred sensations. However, considering we found no suggestive evidence for the mainstream hypothesis that referred sensations are the perceptual correlate of post-amputation S1 plasticity (Collins et al., 2017; Ramachandran, 1992, 1993; Ramachandran & Hirstein, 1998), and given that we were able to successfully provide an alternative mechanistic framework to explain previous reports, our study calls for a general reassessment of this phenomenon and its neural bases. Specifically, while referred sensations might be genuinely and spontaneously experienced by some amputees, the experimental methods used to date to assess this phenomenon clearly contain demand characteristics that at best will contaminate any true effects.

It is well recognized that self-reported phenomena are particularly susceptible to the confounding impact of demand characteristics (Boot et al., 2013; Klein et al., 2012; Szigeti et al., 2021; Turner et al., 1994), yet there have been no attempts to control for these effects in referred sensations testing paradigms. We demonstrate that self-reported referred sensations can be triggered by experimental settings. This is well evidenced not only by the fact that the control groups reported referred sensations in the first place, but also, more directly, by a greater tendency across groups to report referred sensations when they were given the suggestion that these sensations were more likely to occur (‘High’ expectation condition). Expectancy-mediated changes in self-reported experiences can be driven by simple compliance or genuine changes in perception. Although our data does not allow us to dissociate genuine perceptual changes from compliance effects, the observation that congenital one-handers reported referred sensations also on their missing hand, on which touch had never been experienced, and thus no sensations can truly be referred to, suggests that behavioural compliance may have played a prominent role in our results.

The lack of control for demand characteristics poses serious limitations to the interpretation of previous accounts of referred sensations, as well as of other related phenomena that solely rely on self-assessed outcomes without accounting for both experimenter and participants' expectation (e.g., PLP treatment (Ortiz-Catalan et al., 2016)). It is important to note that our findings do not rule out the spontaneous occurrence of referred sensations in selected amputees. Indeed, one participant reported experiencing referred sensations from the neck in his daily life (Amp17 in Table 1) and another (Amp05) reported a “classical” pattern of face-elicited referred sensations in our study (though this did not translate in increased face-hand shared representation or face remapping in S1 missing hand region, as highlighted in Figs. 2D and S3, respectively). We also did not stimulate the entire skin surface, and thus cannot rule out that some potential ‘hot spots’ of referred sensations may have been missed. However, our findings clearly show that this phenomenon can be induced by suggestion and expectation. Insofar as referred sensations reports were plausibly driven, or augmented, by such confounds in previous research, our findings call into question the origins, prevalence and even validity of this perceptual phenomenon. We therefore conclude that previous accounts of referred sensation reports cannot provide a solid perceptual foundation for theories about functional brain reorganisation (Ramachandran & Hirstein, 1998) or for novel efforts to create ecological tactile feedback interfaces for prosthetic limbs (Wijk et al., 2019; D'Alonzo, Clemente, & Cipriani, 2015). We hope that our findings will promote greater consideration of experimental demand characteristics in future research on this and other anomalous perceptual phenomena.

## Contributors

E.A., D.B.T, D.M. and T.R.M. conceived the study. S.K recruited the patients. E.A., M.K. and D.M. collected the data. E.A. and D.M. analysed the data. E.A., D.M. and T.R.M. wrote the manuscript with inputs from all co-authors. T.R.M. secured funding.

## Data availability statement

Anonymised data, materials and code are available at https://osf.io/ygbd5/.

## Open practices

The study in this article earned Open Material badge for transparent practices. The materials used in this study are available at: https://osf.io/ygbd5/.

## Declaration of competing interest

None.
