# Supplementary material for: Reassessing referral of touch following peripheral deafferentation: The role of contextual bias
Source: Cortex. 2023 Oct;167:167–77. doi: 10.1016/j.cortex.2023.04.019 (PMC11139647; doi:10.1016/j.cortex.2023.04.019)
Supplement: Multimedia component 1 [file mmc1.docx]

**SUPPLEMENTAL INFORMATION**

**EXTENDED BEHAVIOURAL METHODS**

**Referred sensation task instructions**

*“In this task you will receive several kinds of stimuli from the vibrotactile stimulators placed on your body. They will feel as gentle vibrations on your skin. Some of these stimulations have been proven to stimulate a newly discovered type of nervous fibres. By doing so, they are able to evoke tactile sensations not only on the actually stimulated area of skin but also on regions of the body further away, and in particular, at the most distal extremities such as the hands or phantom hands. Indeed, due to the configuration of the peripheral nervous system, the hands are the sites at which the dual sensation most likely occurs. In this study we are interested in investigating whether there are sites on your body more responsive to this stimulation, meaning sites that when stimulated are more likely to give rise to these secondary more subtle sensations on the hands. The other types of stimulation stimulate alfa and beta fibres and convey classical information about touch to your brain, without being able to evoke other secondary sensations. On each trial, you will first be cued to which kind of stimulation is coming. Thus, a red circle on the monitor indicates that in the following trial you will receive special stimulation, i.e. the “special” one capable of causing secondary subtle sensations in your hands. A grey circle will appear on the monitor if in the following trial you will receive the classical stimulation. After the coloured circle, you will receive the vibrotactile stimulus. The computer will then ask you whether you felt only one sensation (on the stimulated body site) or more than one. Please press the left pedal to respond that you only felt one and the right pedal to respond that you felt more than one. Please take your time to respond, as it usually takes a while for this interference process to happen. If you responded that you only felt one, you will start the following trial. If you responded that you felt more than one, another screen will appear asking whether you felt the secondary sensation on the left or right hand. Please respond with the left pedal if you felt it in the left or phantom hand and with the right pedal if you felt it in the right hand. The following trial will then start, and the process will be repeated.”*

**Participants’ demographic details**

**Table 1**


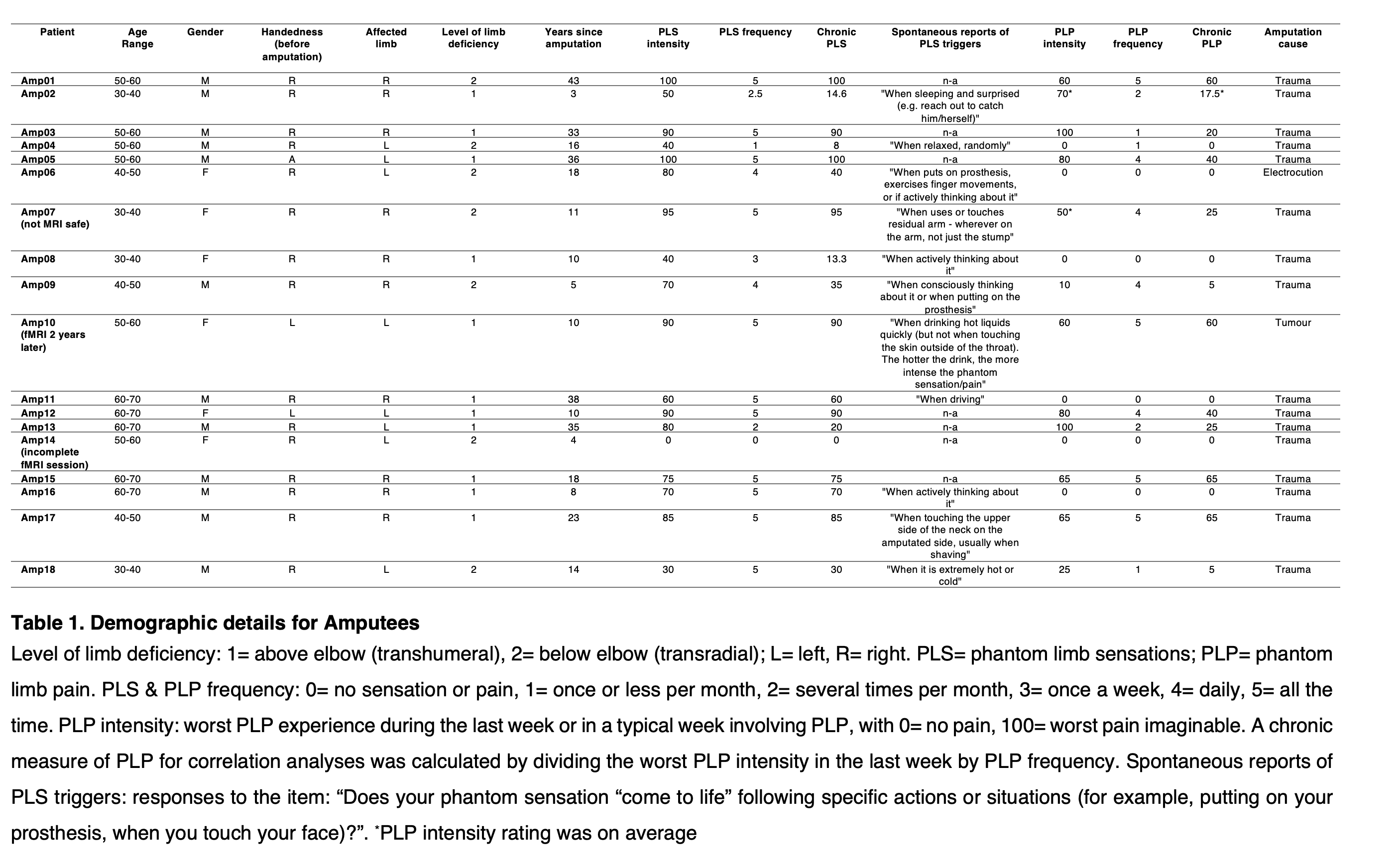


**EXTENDED BEHAVIOURAL RESULTS**

**Referred sensations evoked by non-facial body-parts**

While referred sensations were originally reported to be evoked by the face and residual arm on the phantom hand [1-4] later reports also included other body-parts, including the intact hand and arm, and the feet [5-9]. Here we examined whether participants across groups tended to report more referred sensations on their phantom/missing/non-dominant relative to their intact/dominant hand following stimulation of non-facial sites (i.e. (Intact/Dominant Arm, Intact/Dominant Hand, Foot Ipsilateral and Contralateral to missing/non-dominant hand – 48 trials). Note that here the Residual Arm site was excluded, due to its distinct potential mechanism for inducing referred sensations (via peripheral reinnervation, see Introduction). We found that non-face evoked referred sensations were not reported more frequently on the phantom/missing/non-dominant rather than on the intact/dominant hand in Amputees (N=12) (Z=53.5, p=.265, r_B_=.372, BF_10_=.519), One-handers (N=7) (Z=7.5, p=.310, r_B_=-.464, BF_10_=.499), and Two-handers (N=9) (Z=25.5, p=.326, r_B_=.417, BF_10_=.647). Moreover, no significant group difference emerged (*X^2^*=1.822, p=.402), with Amputees’ phantom lateralised non-face evoked referred sensations not significantly different from One-Handers’ (U=56, p=.249, r_B_=.333, BF_10_=.666) or Two-handers’ (U=51.5, p=.885, r_B_=-.046, BF_10_=.415), and no significant differences between One-handers and Two-handers (U=42.5, p=.262, r_B_=.349, BF_10_=.669)(Figure S1).

**
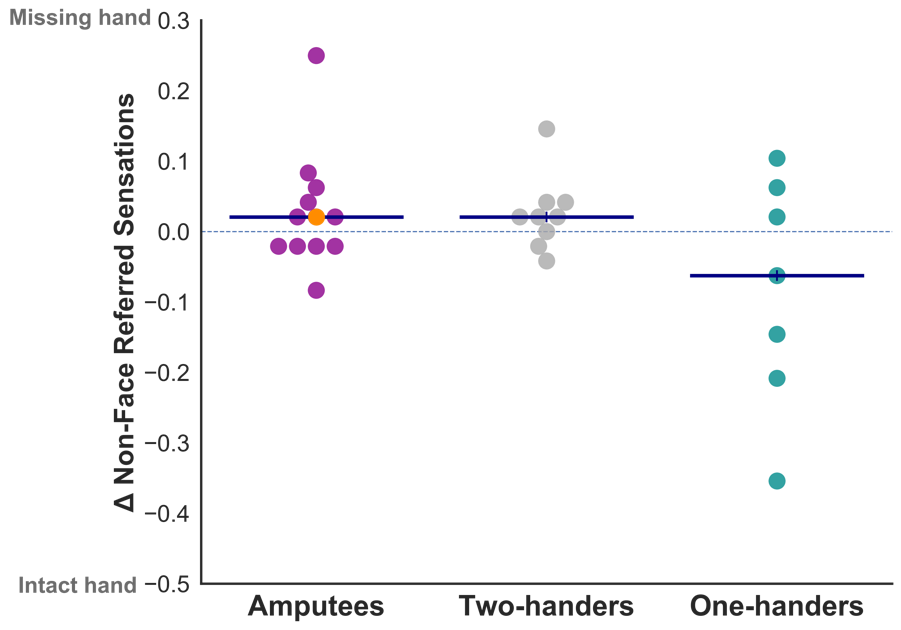
**

**Figure S1.** **Lateralised referred sensations across the non-facial stimulation sites**. Scores are calculated by subtracting the proportion of referred sensations reported on the intact/dominant hand from the proportion of responses on the phantom/missing/non-dominant hand, in Amputees, One-handers and Two-handers, respectively. Participants reporting zero referred sensations across these 48 trials are excluded (52% of total sample). Each dot represents one participant, horizontal blue lines represent group medians. Participants Amp05 is highlighted in orange.

**EXTENDED FMRI RESULTS**

**Remapping in S1 missing-hand area**

To gain further insights into the hypothesized link between referred sensations and S1 remapping, we assessed average activity levels evoked by movement of multiple body-parts in the missing/non-dominant hand area. Compared to Two-handers (N=22), increased activity levels (i.e., remapping) were found in the missing-hand area of One-handers (N=19) (U=86, p<.001, r_B_=-.589), with only a marginal difference between Two-handers and Amputees (N=17) (t_(37)_=-1.928, p=.062, d=-.623, BF_10_=1.315), resulting in a significant interaction between Groups and Hemispheres (F_(2,54)_=8.753, p<.001, 𝜂^2^=.039; ﻿non-parametric equivalent: *X*^2^=10.497, p=.005; Figure S2C). No difference in activity levels in the deprived hemisphere was found between Amputees and One-handers (U=198, p=.257, r_B_=.226, BF_10_=.506).


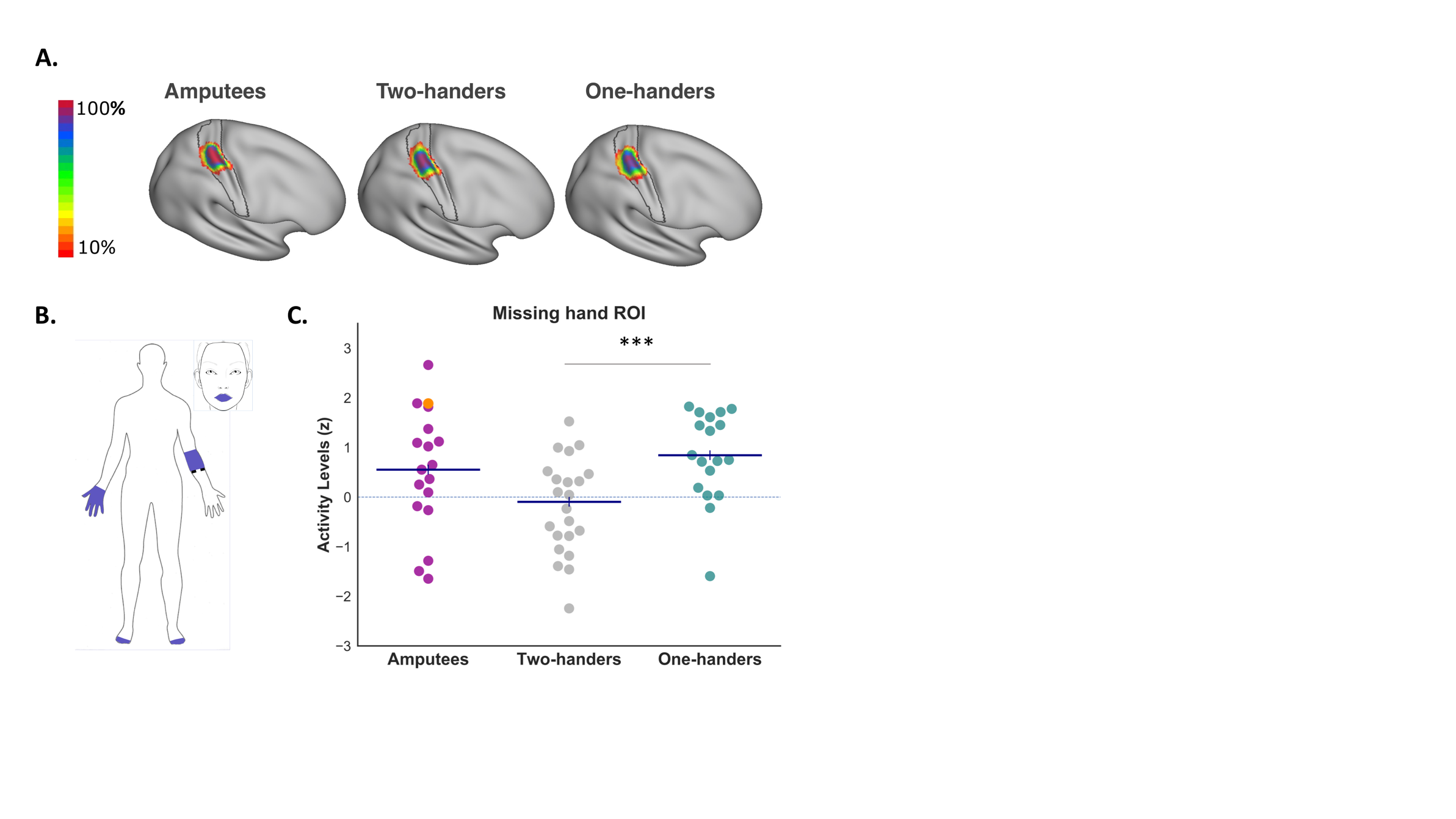


**Figure S2. Remapping in S1 missing-hand area.** **A)** Inter-participant consistency maps for the missing/non-dominant hand S1 regions of interest (ROIs) across the three groups. The colour code represents the number of participants with overlapping ROIs in standard MNI space. The black contour shows the anatomical delineation of S1 used for ROI definition. **B)** Body-parts included in the fMRI body task. **C)** Average (median) BOLD activity levels in the missing/non-dominant hand ROI, evoked by movement of the body-parts shown in B. Each dot represents a participant, horizontal blue lines represent the group medians. Note that, overall, increased remapping can be qualitatively observed in C) for participant Amp05 (highlighted in orange), who reported high rates of phantom referred sensations (Figure 1). ***p < 0.001

These group differences are qualitatively, though clearly, distinguishable from the even inter-group profile observed for the referred sensations reports. In other words, the non-significant group difference for reported sensations on the phantom hand is unlikely to be due to lack of remapping (see below for a replication of these and subsequent results using samples matched to the referred sensation task analyses). In addition, no significant correlation was found between fMRI activity levels in the missing-hand area and chronic PLP in Amputees (N=17, r_Tau_=.103, p=.582, BF_10_=.360).

**Body-parts undergoing S1 remapping**

We then assessed which body-parts were driving the increased activity in the missing-hand area (non-dominant in Two-handers) shown in Figure S2. Since we were considering the contributions of five body-parts, we adjusted our significance (alpha) levels to 0.01.


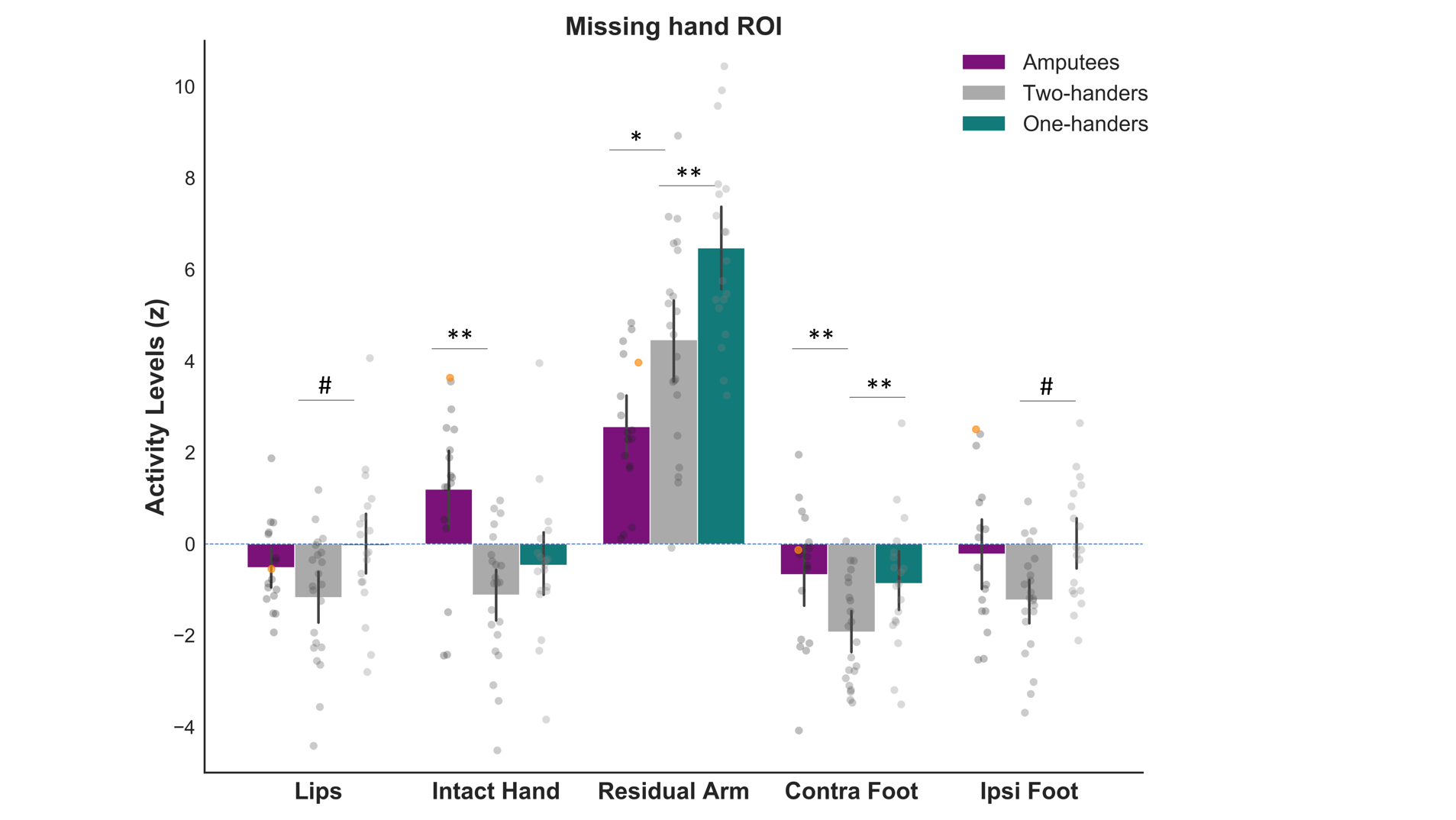


**Figure S3.** **Breakdown of the fMRI activity evoked by different body-parts in the missing-hand area.** Average BOLD activity levels in S1 missing/non-dominant hand area, evoked in each group by movement of the Lips, Intact/Dominant hand, Residual/Non-dominant Arm, Foot Contralateral and Ipsilateral to the missing/non-dominant hand. Each grey dot represents one participant. Participant Amp05 (who reported high rates of referred sensations in the behavioural task – see Figure 1) is highlighted in orange. Asterisks indicate significant group differences: # trend, *p < 0.05, **p ≤ 0.005

Compared to Two-handers (N=22), Amputees (N=17) exhibited increased activity for the intact Hand (t_(37)_=-4.422, p<.001, d=-1.428) and decreased activity for the residual Arm (t_(37)_=2.999, p=.005, d=.969). No increased activity was found in Amputees for the Lips (t_(37)_=-1.664, p=.105, d=-.537, BF_10_=.920). We also found a significant increase in activity for the Foot of the intact side (t_(37)_=-2.978, p=.005, d=-.962) but not for the Foot of the missing side (t_(37)_=-2.242, p=.031, d=-.724, BF_10_=2.137). Taken together, we only find clear and consistent contribution from the intact hand to the identified group differences in activity profiles involving Amputees.

We also considered differences between the congenital One-handers and the Two-handers. Compared to Two-handers (N=22), One-handers (N=19) showed increased activity for the residual Arm (t_(39)_=-2.969, p=.005, d=-.930) and the Foot of the missing side (t_(39)_=-3.238, p=.002, d=-1.014). In addition, trends for increased activity were found for the Lips (t_(39)_=-2.472, p=.018, d=-.774) and the Foot of the intact side (t_(39)_=-2.653, p=.011, d=-.831). No significant differences were found for the intact Hand (t_(39)_=-1.384, p=.174, d=-.433, BF_10_=.652). Taken together, these results indicate that residual arm is the prominent driver of observed group differences in activity levels involving One-handers.

**Sub-sample analysis based on referred sensations reports**

Results in figure 2D and above are reported independent of individual participant’s responses in the behavioural task (i.e., all participants), regardless of whether they reported referred sensations or not (for statistical power considerations). Here, we considered whether we can find evidence for group differences in activity levels while specifically focussing on the sub-set of individuals in each group who reported experiencing referred sensations during the behavioural task. Compared to Two-handers (N=14), both Amputees (N=12) (t_(24)_=-2.707, p=.012, *d*=-1.065) and One-handers (N=12) (U=18, p<.001, *r_B_*=-.786) showed increased activity levels in the missing-hand area (i.e., remapping), resulting in a significant interaction between Groups and Hemispheres (F_(2,34)_=5.604, p=.008, 𝜂^2^=.045; age as covariate; ﻿non-parametric equivalent: *X*^2^=11.676, p=.003). No difference in activity levels in the missing-hand area was found between Amputees and One-handers (U=82, p=.590, *r_B_*=.139, BF_10_=.419). No significant correlation was found between fMRI activity levels in the missing-hand area and chronic PLP in Amputees (N=12, r_Tau_=.162, p=.482, BF_10_=.461).

We then assessed which body-parts were driving this increased activity in the missing-hand area. As above, alpha levels were adjusted to 0.01 to correct for the five comparisons across body-parts. Compared to Two-handers (N=14), Amputees (N=12) showed increased activity levels for the intact Hand (t_(24)_=-4.032, p<.001, d=-1.586) and for the Foot on the intact side (U=31, p=.005, r_B_=-.631). A trend for increased activity was also found for the Foot on the missing side (t_(24)_=-2.690, p=.013, d=-1.058, BF_10_=4.274). No differences were found for the Lips (t_(24)_=-1.246, p=.225, d=-.490, BF_10_=.640) or the residual Arm (t_(24)_=1.022, p=.317, d=.402, BF_10_=.533). Compared to Two-handers (N=14), One-handers (N=12) exhibited increased activity for the residual Arm (t_(24)_=-3.802, p<.001, d=-1.496) and for the Foot of the missing side (t_(24)_=-2.875, p=.008, d=-1.131). No differences were found for the Lips (t_(24)_=-2.285, p=.031, d=-.899, BF_10_=2.245), for the Foot of the intact side (U=50, p=.085, r_B_ =-.405, BF_10_=1.067) or for the intact Hand (U=59, p=.212, r_B_=-.298, BF_10_=0.643).

Finally, we also compared the representational dissimilarity between activity patterns evoked by face and the contralateral thumb movement and we found no significant differences between Amputees and Two-handers (F_(1,17)_=.176, p=.680, 𝜂^2^=.009), and no interaction with the Hemisphere (F_(1,17)_=.008, p=.929, 𝜂^2^<.001). Follow-up comparisons revealed no significant difference between Hemispheres in Amputees (t_(8)_=.316, p=.760, d=.105, BF_10_=.336), as well as no group difference in the dissimilarities observed in the missing/non-dominant hand area (t_(17)_=-.405, p=.690, d=-.186, BF_10_=.429)).

**REFERENCES**

1. [Halligan PW, Marshall JC, Wade DT. Sensory disorganization and perceptual plasticity after limb amputation: a follow-up study. *Neuroreport* 1994;5:1341–5.](http://paperpile.com/b/IOCg7e/i68E)
2. [Ramachandran VS, Rogers-Ramachandran D, Stewart M. Perceptual correlates of massive cortical reorganization. *Science* 1992;258:1159–60.](http://paperpile.com/b/IOCg7e/Oh7C)
3. [Ramachandran VS. Behavioral and magnetoencephalographic correlates of plasticity in the adult human brain. *Proc Natl Acad Sci U S A* 1993;90:10413–20.](http://paperpile.com/b/IOCg7e/j0ER)
4. [Borsook D, Becerra L, Fishman S, *et al.* Acute plasticity in the human somatosensory cortex following amputation. *Neuroreport* 1998;9:1013–7.](http://paperpile.com/b/IOCg7e/eiOI)
5. [Grüsser SM, Mühlnickel W, Schaefer M, *et al.* Remote activation of referred phantom sensation and cortical reorganization in human upper extremity amputees. *Exp Brain Res* 2004;154:97–102.](http://paperpile.com/b/IOCg7e/QdyQ)
6. [Grüsser SM, Winter C, Mühlnickel W, *et al.* The relationship of perceptual phenomena and cortical reorganization in upper extremity amputees. *Neuroscience* 2001.doi:](http://paperpile.com/b/IOCg7e/1imG)[10.1016/S0306-4522(00)00491-7](http://dx.doi.org/10.1016/S0306-4522(00)00491-7)
7. [Andoh J, Diers M, Milde C, *et al.* Neural correlates of evoked phantom limb sensations. *Biol Psychol* 2017;126:89–97.](http://paperpile.com/b/IOCg7e/W941)
8. [Knecht S, Henningsen H, Elbert T, *et al.* Reorganizational and perceptional changes after amputation. *Brain* 1996;119(Pt4):1213–9.](http://paperpile.com/b/IOCg7e/FmcQ)
9. [Knecht S, Henningsen H, Höhling C, *et al.* Plasticity of plasticity? Changes in the pattern of perceptual correlates of reorganization after amputation. *Brain* 1998;121(Pt4):717–24.](http://paperpile.com/b/IOCg7e/13UI)
